# Hysterectomy for Uterine Fibromata

**Published:** 1897-05

**Authors:** L. C. Ruter

**Affiliations:** Madison, Fla.


### HYSTERECTOMY FOR UTERINE FIBROMATA.—FATAL TERMINATION.

By L. C. RUTER, M. D., MADISON, FLA.

I saw Mrs. R. for the first time about the first of last February. She was twenty-eight years of age. Mother of two children; had miscarried once or twice. Her youngest child was seven years old. She menstruated regularly with no *excess* and little or no pain. Family history good so far as I could learn. She was anemic, had lost flesh rapidly, and was very nervous. Bowels constipated, digestion poor, no appetite. Temperature, 99½, pulse, 80. She said she suffered constantly with an aching, throbbing pain in her left side, which would radiate up into her back and down her thighs. Upon physical examination I could easily feel a dense, hard mass in her left flank, very tender upon pressure, and seemingly continuous with the uterus. The uterus was not movable in any direction; impossible to pass a sound, could pass it through the cervix with ease, but no further. Her history of the case was about as follows: About one year ago she had severe pain in the lower part of her abdomen with fever and quick pulse. There was a copious discharge from the womb. She was treated for this trouble for about two weeks and got better, but was never well from this time on. After thoroughly examining her, I told her that surgery was all that offered her any chance of relief. I explained the seriousness of the operation, but told her she might live as she was living then her natural lifetime, but that she could never hope to get any better, and the tendency was for her condition to get worse. She took the matter of operation under consideration, and talked it over with her husband and concluded to have the operation done. Having everything carefully arranged, I began the operation at about 11:30 A. M., on April 29th. I was assisted by Drs. C. H. Smith, A. L. Blalock and W. H. Paramore. I made an incision from about two inches below the umbilicus to the symphysis pubis. I introduced my hand and could feel a tumor that was continuous with the uterus. Following it down I found the tubes and ovaries solidly bound down in the pelvis on both sides and everything so completely matted together that all anatomical landmarks were lost.

The part of the tumor which was in the left side seemed to be fibroid in structure, just about the size of the fundus and felt like a continuation of the fundus of the uterus. It was, however, a non-pedunculated fibroid of the fundus. We thought we would try to tie off the ovarian artery on the right side and see if we could loosen up the mass enough to see if it could be removed. We found that tying off a portion of it gave us no advantage whatever as far as adhesions were concerned over that remaining. But we continued carefully to dissect our way down until we got the parts in the right flank loosened up only to find the lower part of the rectum where it passes over the sacrum firmly matted to the mass and at this point, about at the junction of the tumor with the uterus, there was an abscess in the tumor from which pus freely flowed. I think it had already at sometime broken through into the bowel. At any rate the sac was very thin and would soon have broken some place again. I should have said we put the patient in Trendelenberg's position after the first incision. We then examined anteriorly and found the bladder firmly attached in front. It now looked as if we had run onto an impossibility. We held a quick consultation as to whethar it would be the better plan to abandon the operation or finish it at any risk. My colleagues thought it would be the best plan to close up the abdomen and quit. I thought over it for an instant and could not bear the idea of letting the patient come out of the anesthetic, and not only telling her that we could not help her, but there was no chance of her ever being any use to herself or any one else, and that she must continue to suffer and finish it all with death in a short time. I thought under the circumstances we were justified in taking the whole mass out at any risk. So we continued ligating and cutting between the ligatures until we had the right side loose with the tube and ovary of this side attached to the tumor (when I speak of the tumor I mean the tumor, uterus and all), we worked on the left side then until we got the main or larger part of the tumor proper, which was in the left side separated pretty well from its attachments in this side. The tube and ovary under this part of the tumor on this side was a suppurating mass and could not be taken out attached to the tumor, so we had to take what we could get off them and leave the rest, which was bound down so tightly it could not be taken out. We now came down to where it was attached to the bowel. I thought I would go slow and keep my finger tightly pressed against the tumor and break it little by little until I got it separated, but before I had worked long and only making slight pressure I tore a slight rent in the bowel and still did not have it freed. But I still continued trying to separate it until I tore the bowel again, but with this tear the bowel was free. We now had it loose at both sides and behind, and now turned our attention to the anterior part. We found the adhesions to the bladder were not so bad and we soon loosened them, and amputated the uterus at about the internal os. We had been three or four hours at hard, painful, patient work since I made the first incision. The cervix seemed to be all right.

We started to close the rents in the bowel and suture the stump (or cervix) and arrange for drainage, but by this time the patient was sinking and we saw that all of our work had been done in vain. Seeing she would very soon die, we closed the abdomen, and she died in about a quarter or half hour from the time we removed the tumor. To say the termination of this case was painful and discouraging to us, does not express it. She was a brave Christian woman, and put her life in our hands with no hesitation whatever, after deciding to undergo the operation, saying if there was any relief for her she would be glad to get it, but if she died it was all right, she was ready. We were therefore anxious to make a success, but the odds were against us. Examining the tumor after its removal, we found it to be a fibroid, broken down down into a suppurating mass posteriorly. We incised the uterus and found the cavity almost obliterated, and taking it as a whole one would never recognize it as a uterus

I report this case for several reasons:

First. Because, in justice to the profession, we should report our failures.

Second. We often learn as much or more by our failures than by successes.

Third. It concerns a fibroid, with normal menstruation. In this case it was probably due to the small part of the endometrium that was left, not more than a half inch square.

Fourth. It plainly showed that a fibroid may do something besides causing hemorrhage, and letting them alone until the menopause will not always be safe. They sometimes break down as in this case, and when they do it makes a serious condition of affairs.

Fifth. Be careful how you try to separate adhesions from the bowel.

Sixth. I do not wish to discourage any one, but if I ever run upon another case that I have reason to believe is as complicated as this one was, I will close up the abdomen and abandon the operation. Not that I think the patient would be better off, for her life could certainly have been nothing but suffering, ending in a short time in death.

But the chances for success in a case like this are very scant. Unless you and your patient are both willing to make one last effort at any risk, it would be wise to quit the operation in the beginning; that is stop it after you have made the exploratory incision if you find it, as I did in this case, almost an impossibility to complete the operation.

L. C. RUTER.

*Madison, Fla.*

